# Non-coding functions of alternative pre-mRNA splicing in development

**DOI:** 10.1016/j.semcdb.2015.10.018

**Published:** 2015-12

**Authors:** Stefan Mockenhaupt, Eugene V. Makeyev

**Affiliations:** a School of Biological Sciences, Nanyang Technological University, Singapore 637551, Singapore; b MRC Centre for Developmental Neurobiology, King's College London, London SE1 1UL, UK

**Keywords:** A3E, alternative 3′ terminal exon, APA, alternative cleavage and polyadenylation, ARE, AU-rich element, AS, alternative splicing, IR, intron retention, NMD, nonsense-mediated decay, NMTR, nonsense-mediated translational repression, NRE, nuclear retention and elimination, nt, nucleotide, PTC, premature termination codon, RUST, regulated unproductive splicing and translation, uORF, upstream open reading frame, Alternative pre-mRNA splicing, mRNA stability, Translational regulation, mRNA localization, Development

## Abstract

A majority of messenger RNA precursors (pre-mRNAs) in the higher eukaryotes undergo alternative splicing to generate more than one mature product. By targeting the open reading frame region this process increases diversity of protein isoforms beyond the nominal coding capacity of the genome. However, alternative splicing also frequently controls output levels and spatiotemporal features of cellular and organismal gene expression programs. Here we discuss how these non-coding functions of alternative splicing contribute to development through regulation of mRNA stability, translational efficiency and cellular localization.

## 1 Introduction

Eukaryotic genomes contain a large number of intronic sequences that “split” gene-encoded messages at the level of DNA and mRNA precursor transcripts (pre-mRNAs) but are spliced out from the mature mRNAs [1]. A large ribonucleoprotein complex called the spliceosome catalyzes this reaction either co-transcriptionally or following the release of a nascent transcript from the RNA polymerase complex [2], [3], [4], [5].

**Fig. 1:**
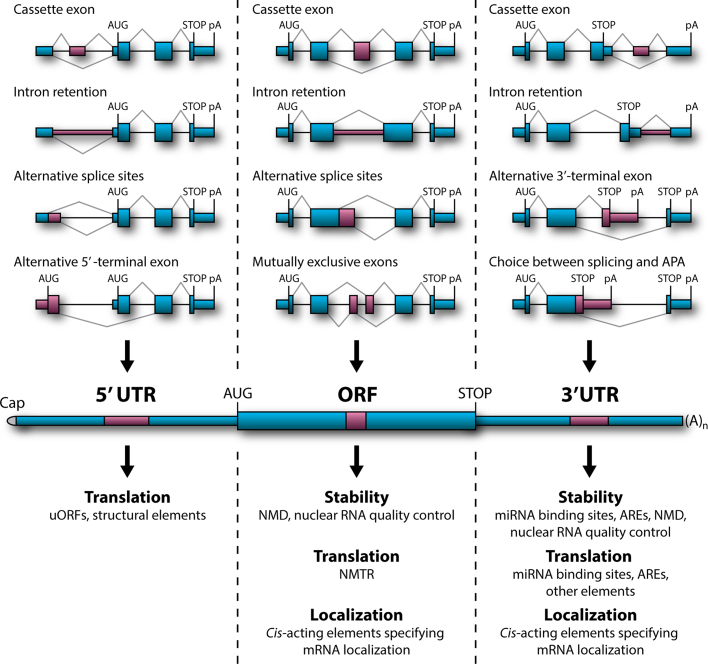
Role of AS in mRNA stability, translational activity and subcellular localization. Top, examples of relevant AS topologies; mid, mature mRNA containing 5′ and 3′UTRs flanking the protein-coding ORF; bottom, downstream regulation outcomes of AS events in the corresponding regions.

Soon after the discovery of split genes [6], [7] it became obvious that some pre-mRNAs can be spliced in more than one way to give rise to distinct mature products [8], [9]. Subsequent studies showed that such alternative splicing (AS) events are extensively controlled by *cis*-regulatory RNA sequences and *trans*-acting splicing factors [3], [10], [11]. Moreover, a number of AS topologies have been described including selection between alternative 5′ or 3′ splice sites, cassette exons, mutually exclusive exons, alternative 5′ or 3′ terminal exons (A5Es and A3Es), intron retention (IR) and a range of combinations between these possibilities [10], [11] (also see Fig. 1).

Recent work demonstrated that more than 90% of intron-containing pre-mRNAs in mammals might undergo AS [12], [13], [14]. What could be biological functions of this widespread regulation? One answer appears to be an effective increase in the coding capacity of the genome. Indeed, ∼21,000 human genes are estimated to give rise to ∼80,000 differentially spliced mRNA species [15] that often encode distinct protein isoforms. Such AS-mediated expansion of proteome complexity might have facilitated progressive evolution of multicellular eukaryotes [16], [17].

However, a large fraction of AS events occurs in the 5′ and 3′ untranslated regions (UTRs) of mRNA flanking the open reading frame (ORF). This has no effect on the polypeptide sequence but instead may modulate other aspects of mRNA behavior. Moreover, mounting evidence suggests that many AS-mediated changes in the ORF sequence can also modify mRNA stability, translational activity and subcellular localization [18], [19], [20], [21], [22], [23]. Here we discuss how such non-coding functions of AS events in the 5′UTR, 3′UTR and ORF-encoding regions contribute to development with a particular focus on recently published examples (see Fig. 1 for a graphical summary).

## 2 The 5′UTR

Eukaryotic gene expression is extensively controlled at the level of mRNA translation and stability and the 5′UTR plays an important part in this regulation [24], [25]. Mature mRNAs can acquire distinct 5′UTR sequences through alternative utilization of transcription initiation sites or 5′-proximal non-coding exons. Mechanisms underlying promoter choice in development have been extensively reviewed elsewhere (e.g., [26]) and we will consider only *bona fide* AS events below.

### 2.1 Upstream ORFs

A widespread mechanism modulating mRNA translational efficiency depends on short upstream open reading frames (uORFs) encoded in the 5′UTR sequence. uORFs tend to reduce translation efficiencies of downstream protein-coding ORFs or destabilize mRNAs through nonsense-mediated decay (NMD) [27], [28], [29], [30] (also see Section 4.1 for further description of NMD). Notably, uORFs are found in up to 50% of mammalian genes and their utilization is frequently regulated by AS [31], [32].

A relevant example is provided by the adiponectin receptor 1 gene (*ADIPOR1*) involved in the regulation of cellular glucose uptake and body size [33]. In one of the ADIPOR1 mRNA isoforms, inclusion of the alternative exon 1c into the 5′UTR leads to the appearance of two translationally repressive uORFs [34]. Notably, exon 1c utilization increases during differentiation of myoblasts into myotubes and correlates with insulin sensitivity thus suggesting a role for this AS event in normal muscle development and the onset of type 2 diabetes.

Similarly, utilization of a non-coding cassette exon can modify the 5′UTR of mRNA encoding human Disc Large Homolog 1 (DLG1), a scaffolding protein expressed in epithelial cells and required for proper cardiovascular development [35]. This exon interferes with DLG1 translation at least in part by introducing a short uORF into the 5′UTR sequence.

Intron 1 in the 5′UTR of mRNA encoding chick proinsulin, an insulin precursor essential for proper development and metabolism, is increasingly retained during embryogenesis, thus reducing mRNA translational activity and lowering proinsulin production [36]. This intron-retaining isoform is up-regulated during transition from gastrulation to organogenesis in the heart but not the pancreas, thus contributing to establishment of normal proinsulin expression patterns. A similar IR event in the mouse proinsulin pre-mRNA also occurs in development but has no detectable effect on mRNA translation [37]. This difference might be due to the presence of multiple uORFs in the chick but not the mouse intron 1.

Surfactant protein A (Sp-A) is a surface-coating protein involved in immune response and normal functioning of the lung [38]. The human SP-A locus comprises two functional genes called *SP-A1* and *SP-A2* and both of them can generate a number of 5′UTR variants through combinatorial utilization of four alternative exons [38], [39], [40]. At least one of these mRNA species, the Sp-A1 ACD’ isoform, contains an uORF that has been shown to dampen protein production [40].

Finally, 15 different 5′UTR variants have been identified for the glucocorticoid receptor (GR) mRNA in humans and 11 and 10 in rat and mouse, respectively [41]. These arise by splicing of a constitutive 5′-most exon 1s with variable downstream exons controlling GR translation through the uORF-dependent mechanism and regulating mRNA stability in an uORF-independent manner.

### 2.2 Secondary structure elements

Another common strategy depends on AS-regulated changes in the 5′UTR secondary structure. Developmentally controlled retention of intron 1 in transcripts encoding the spliceosome assembly factor RNP-4F generates two major 5′UTR variants in *Drosophila* [42]. The retained 177 nt-long sequence and parts of the downstream exon 2 fold into a stable stem-loop structure facilitating translation of the downstream ORF in the mRNA isoform with the longer 5′UTR variant. Interestingly, RNP-4F protein appears to promote splicing of the retained intron 1 within its own pre-mRNA, which may function as a negative feedback maintaining RNP-4F expression homeostasis [43].

The *ZIF2* gene in *Arabidopsis* generates a fully spliced mRNA called ZIF2.1 and an alternative isoform, ZIF2.2, containing an unspliced intron in its 5′UTR [44]. Although both isoforms encode the same transporter protein required for zinc (Zn^2+^) tolerance, ZIF2.2 produces a stronger protective effect in transgenic plants than ZIF2.1. This effect is mediated by Zn^2+^-dependent activation of translation through a stable stem-loop element present in the 5′UTR of ZIF2.2 but not ZIF2.1. Importantly, increased concentrations of Zn^2+^ dramatically stimulate expression of the longer ZIF2.2 isoform thus providing a mechanism for adaptive environmental stress response.

In another example, human proinsulin 5′UTR has two closely positioned alternative 5′ splice sites. These give rise to two mRNA products that differ in length by 26 nt and have distinct secondary structures of the 5′UTR [45]. Interestingly, the longer isoform also shows a higher translational efficiency and its expression in the pancreas is stimulated by glucose [45]. Thus, metabolic cues can fine-tune pancreatic proinsulin production through AS of the 5′UTR.

## 3 The 3′UTR

This region tends to play a critical part in mRNA stability, translational efficiency and localization and contain developmentally important *cis*-elements. Similar to the 5′UTR defined through transcription start site choice and 5′-terminal splicing patterns, two distinct molecular processes can modulate 3′UTR composition: AS and alternative pre-mRNA cleavage and polyadenylation (APA). We will discuss the role of splicing in this process referring the reader to several excellent reviews on mechanisms and functional consequences of APA [46], [47], [48].

### 3.1 microRNA-binding sites

microRNAs (miRNAs) are ∼22-nt long endogenously encoded regulators of mRNA translation and stability that play important roles in essentially all aspects of development [49], [50], [51]. miRNAs function through sequence-specific interaction with their cognate binding sites located predominantly in the 3′UTRs of mRNA targets. Importantly, availability of approximately one third of miRNA binding sites may be controlled by 3′UTR-specific AS [52].

For example, divalent metal transporter 1 gene (*DMT1*) contains two alternative 3′ terminal exons (A3Es), exons 16 and 17 [53]. This produces two different mRNA isoforms encoding protein isoforms either containing iron response element (DMT1 + IRE; exon 16 inclusion) or lacking this element (DMT1-IRE; exon 17 inclusion). Exon 17 carries a binding site for miRNA let-7d, which limits the expression of the DMT1-IRE but not the DMT1 + IRE protein isoform in erythroid precursor cells. During erythroid differentiation, let-7d is naturally down-regulated thus allowing DMT1-IRE to become more prevalent than DMT1 + IRE. Notably, let-7d overexpression results in impaired erythroid cell differentiation due to accumulation of iron in the endosomes, consistent with deregulation of the DMT1 isoform ratio.

Transcripts encoding methyl-CpG binding protein MBD2 provide another example of functional coupling between AS and the miRNA pathway [54]. In this case, selection between two A3Es gives rise to MBD2a and MBD2c protein isoforms. Both isoforms can interact with promoter regions of the stem cell-specific genes *OCT4* and *NANOG.* However, the downstream effect of this interaction is isoform-specific: MBD2a promotes human pluripotent stem cell differentiation, whereas MBD2c activates OCT4 and thus reinforces the stem cell status. Interestingly, OCT4 stimulates utilization of the MBD2c-specific A3E through splicing factor Srsf2/Sfrs2/SC35. On the other hand, OCT4 mediates production of microRNAs from the miR-302 family, which target the MBD2a- but not the MBD2c-specific 3′UTR. These positive feedback mechanisms facilitate stem cell maintenance.

Regulated A3E choice also orchestrates miRNA regulation of the sarcoplasmic/endoplasmic reticulum Ca^2+^-pump Serca2 during differentiation of ESCs into cardiomyocytes [55]. Developmental transition from the ESC-specific isoform Serca2b to the cardiomyocyte-specific isoform Serca2a is essential for proper cardiac function. Several miRNAs including miR-200b and miR-214 target the 3′UTR of Serca2b but not Serca2a and thus contribute to the mutually exclusive expression of the two isoforms in the heart and other tissues.

Similarly, the anti-apoptotic *Bcl2* gene gives rise to two mRNA isoforms: Bcl2α containing the 3′-terminal exon 3 and encoding the fully functional Bcl2 protein and Bcl2β terminated at an internal APA competitor of the terminal exon 3 and encoding a truncated isoform [56]. The 3′UTR of Bcl2α but not Bcl2β is targeted by miR-204 in human cells leading to selective down-regulation of the former isoform in the presence of this mRNA. As an additional layer of regulation, the RBP Tra2β can bind to the miRNA target site and rescue Bcl2α from miR-204 mediated repression. Notably, reduced Tra2β or elevated miR-204 levels can decrease Bcl2α expression and trigger apoptosis.

### 3.2 AU-rich elements

RNA-binding proteins (RBPs) control virtually all aspects of mRNA behavior in the cell [57], [58]. An archetypal example of this regulation mode is 3′UTR-enriched AU-rich elements (AREs) that modulate stability and translational activity of multiple mRNA targets by recruiting corresponding RBPs [59], [60], [61], [62]. Similar to miRNA binding sites, AREs can be regulated by AS [63].

For instance, the choice between three A3Es in mRNA of human parathyroid hormone-related protein (PTHrP) involved in bone development gives rise to 139, 173 or 141 amino acid (aa)-long protein species [64]. Of the three mRNA isoforms, the one encoding the 141 aa-long protein is the least stable due to the presence of AREs in its 3′UTR. However, exposure to Tgfβ results in stabilization of this mRNA through a yet-to-be identified mechanism potentially regulating PTHrP expression in development.

An opposite effect has been reported for mRNAs encoding β-catenin, an important component of the Wnt signaling pathway [65]. In this case, three different 3′UTRs are generated through alternative use of intron 15 and exon 16A and all of these variants contain AREs [66]. Surprisingly, the AREs appeared to stabilize the two shorter isoforms containing one or two exon–exon junctions within their 3′UTRs while having no effect on their longer counterpart. Cellular β-catenin protein levels showed the strongest correlation with expression levels of the shortest and the most stable isoform hinting at possible functional importance of the ARE-dependent stabilization.

### 3.3 Other elements regulating mRNA stability and translation

In several cases, AS-regulated 3′UTRs have been shown to control protein expression levels through poorly understood mechanisms. For instance, inclusion of exon 23 into integrin α7 (Itga7) mRNA gives rise to a protein with altered C-terminus and concomitantly extends the 3′UTR sequence [67]. The exon 23-containing isoform is specifically expressed in differentiated myogenic cells but not in proliferating precursors where it appears to be destabilized by a yet-to-be identified mechanism.

TDP-43 protein implicated in amyotrophic lateral sclerosis and frontotemporal dementia auto-regulates its own expression by a complex change in the 3′UTR involving APA and intron splicing [68]. Notably, the mRNA isoform produced in the presence of increased amounts of TDP-43 protein is retained and subsequently degraded in the nucleus thus providing a mechanism for TDP-43 homeostasis. This regulation is evocative of examples discussed in Section 4.3; however, additional studies will be required to fully understand its molecular details.

An example of translational regulation is the mRNA of conserved polarity factor Par-5 directing asymmetrical cell division and subsequent cell type specifications in one-cell *C. elegans* embryos [69]. Par-5 protein levels are regulated by alternative processing of the 3′UTR sequence. The longer 3′UTR isoforms 1 and 2, exhibit higher translational efficiencies than the shorter isoform 3 thus pointing to a presence of translation activation element(s) in the 3′UTR extension. Although molecular mechanisms regulating Par-5 isoform translation are presently unknown, this circuitry is regulated during development and essential for robust polarization of the embryo.

CPEB1 is an RNA-binding protein regulating mRNA polyadenylation status and translation in the cytoplasm and essential for proper oocyte development [70], [71]. CPEB1 can also localize to the nucleus where – besides its other activities – it can regulate AS by inhibiting RNA recruitment of the core spliceosome component U2AF65. An important consequence of this regulation is a switch to an alternative 3′UTR in the mRNA encoding the mitotic checkpoint protein Bub3 [72]. This has been shown to stimulate Bub3 mRNA translation [72].

Neurexins (Nrxn) are highly diverse pre-synaptic transmembrane proteins that are involved in axon guidance and synapse morphogenesis and function [73]. There are three Nrxn genes, Nrxn1-3, each containing two alternative promoters giving rise to α and β isoforms. Moreover, combinatorial use of multiple alternative exons allows these genes to produce thousands of distinct protein isoforms, which may provide a molecular code for synaptic connectivity within neuronal networks [74]. However, some AS events including the alternative exon 24a in the Nrxn3α 3′UTR transcripts have been shown to mediate translational repression [73]. It is tempting to speculate that this might affect neuronal network structure by fine-tuning Nrxn3α expression levels.

### 3.4 Elements modulating mRNA localization in the cell

Targeting mRNA to specific locations within a cell affords spatially restricted protein synthesis and has important functions in development [75], [76], [77], [78], [79], [80], [81], [82]. *Cis*-elements specifying mRNA localization often reside in the 3′UTR [83] and thus can be regulated by AS in this region. Indeed, mRNA of glial fibrillary acidic protein (GFAP) has several splice variants, of which GFAPδ uses a different A3E compared to the predominant GFAPα isoform [84]. Interestingly, a larger fraction of GFAPα transcripts localizes to astrocyte protrusions as compared to GFAPδ and this difference can be attributed to differences in the two 3′UTR sequences.

Similarly, three alternative 3′UTRs have been described for the mRNA of human plakophilin 4 protein (Pkp4; p0071) involved in the assembly of adherens junctions and desmosomes. Interestingly, the nature of the 3′UTR determined whether the mRNA localized to cellular protrusions during cell migration and only isoforms using exon 21 as their A3E showed this behavior [85]. This effect appeared to be cell type-specific thus hinting at possible involvement of additional factors.

## 4 The ORF region

The main ORF function is to provide a template for ribosome-mediated protein production. However, this region can additionally encode regulatory elements along with the amino-acid sequence [86]. Below, we discuss how utilization of such non-coding features can be regulated by AS.

### 4.1 Nonsense-mediated decay

An extensively studied strategy for ORF-encoded regulation of mRNA behavior, involves functional coupling between AS and cytoplasmic mRNA quality control mechanism called nonsense-mediated decay (NMD). NMD machinery detects and eliminates mRNAs containing premature translation termination codons (PTCs). In mammals, NMD is thought to be mediated by interplay between exon junction complexes (EJCs) deposited in the nucleus upstream of most exon-exon splice junctions and the translation termination complex (TTC) along with a host of additional factors [87], [88], [89], [90]. EJCs remain attached to the mRNA during its export from the nucleus to the cytoplasm but are dislodged by the ribosomes during the pioneering round of translation. Since most mammalian exon–exon junctions occur within or relatively close to the ORF region, a typical translationally active mRNAs rapidly loses its complement of EJCs in the cytoplasm and becomes immune to NMD. However, a PTC appearing through mutations, splicing errors or AS, limits the sequence window accessible to translating ribosomes such that one or several EJCs positioned downstream of the truncated ORF remain attached to the mRNA and trigger mRNA decay upon their interaction with the TTC.

Although initially identified as a surveillance mechanism intercepting aberrant mRNAs, NMD additionally functions in combination with AS to orchestrate programmed changes in gene expression levels. This mechanism (AS-NMD; also called RUST from “Regulated Unproductive Splicing and Translation”) has been described in several recent reviews [19], [20], [21], [22], [23], [91]. We will therefore discuss only two AS-NMD scenarios recurring in developmental contexts: control of master regulators of cellular RNA metabolism and coordinated regulation of cell type-specific gene batteries.

The former scenario often involves negative feedback loops mediated by interaction of an AS factor with its own pre-mRNA and repression of an alternative exon essential for ORF integrity. For example, polypyrimidine-binding protein 1 (Ptbp1/PTBP/hnRNP I) promotes skipping of the ORF-maintaining exon 11 in its primary transcripts which ensures homeostasis of this RNA-binding protein [92] (see [19], [22], [23] for other examples). On the other hand, SR proteins and other splicing factors capable of splicing activation maintain their expression homeostasis by stimulating utilization of specialized (“poison”) alternative exons encoding an in-frame PTC or shifting the ORF to expose a PTC in downstream constitutive exons [19], [93].

AS-NMD can additionally mediate cross-regulation between distinct RNA-binding proteins (see e.g., [19], [22], [23]). In a recently published example, STAR (signal transduction and activator of RNA) domain-containing splicing factor Slm2 has been shown to repress expression of its paralog Slm1, thus ensuring correct AS in mouse hippocampus [94]. Underscoring importance of this regulation modality in development, most splicing factors controlled by AS-NMD contribute to establishment and maintenance of cell type-specific transcriptomes by coordinating large groups of AS events. Moreover, many ORF-maintaining and poison exons in splicing factor genes are conserved across species and occasionally different eukaryotic kingdoms [91], [93], [95], [96], [97], [98].

Apart from splicing factors, AS-NMD is known to regulate many other genes with important developmental and physiological functions. One particularly interesting scenario is coordinated AS-NMD regulation of cell type-specific genes. This mechanism is used to co-regulate at least 86 functionally related genes in developing granulocytes [99] and a sizeable fraction of differentiation-specific genes during erythropoiesis [100]. Notably, a recent transcriptome deep-sequencing effort uncovered 1014 AS-NMD exons utilized in mouse cortex and showing a degree of evolutionary conservation [101]. Interestingly, besides the expected enrichment for regulators of RNA metabolism, a substantial fraction of genes containing these exons encoded chromatin regulators. This finding might hint at a large-scale AS-NMD coordination of functionally linked genes in developing brain. In line with this notion, neuron-specific RBP NOVA has been shown to have several important AS-NMD targets [102]. Similarly, natural down-regulation of Ptbp1 in developing neurons is known to promote expression of critical post-synaptic components (e.g., Gabbr1 and PSD-95) and reduce expression of non-neuronal markers through AS-NMD [103], [104], [105].

### 4.2 Nonsense-mediated translational repression

A subset of PTC-containing mRNAs may escape NMD and become targets of a distinct cytoplasmic quality control known as nonsense-mediated translational repression (NMTR; [106]). Although molecular mechanisms underlying this process are not understood completely, PTC recognition in some mRNAs requires downstream EJCs [107]. Moreover, phosphorylation of a key NMD key component, Upf1 protein, upon PTC recognition is known to trigger translational repression prior to delivery of the targeted mRNA to the decay machinery [108]. It is therefore possible that NMTR represents an “incompletely executed” NMD program. However, NMTR could also coopt distinct molecular mechanisms, e.g., acquisition of repressive *cis*-elements in the extended 3′UTR sequence [109].

Similar to AS-NMD, combination of AS and NMTR may contribute to regulation of gene expression. For example, pre-mRNA of pro-apoptotic Bak1 protein contains the 20-nt long cassette exon N skipped in non-neuronal cells but activated in neurons. Inclusion of this exon shifts Bak1 ORF and leads to the appearance of a PTC. Notably, the PTC containing mRNA does not appear to be targeted by NMD but its translation is repressed in part due to the presence of a downstream EJC [110], [111]. Similarly, expression of the actin-related transcriptional repressor of muscle-specific genes Arp5 is controlled by a choice between two alternative 3′ splice sites (ss) in exon 7 [109]. Preferential utilization of the downstream 3′ss in differentiated smooth muscle cells generates a PTC and down-regulates Arp5 protein levels via both NMD and NMTR. Interestingly, a number of alternatively spliced mRNA isoforms containing PTCs appear to escape NMD in human HeLa cells, which suggests that the repertoire of AS-NMTR targets might be substantially wider than currently thought [112].

### 4.3 RNA quality control in the nucleus

Quality of eukaryotic mRNAs is also controlled in the nucleus [113], [114], [115]. An important nuclear QC mechanism limits export of incompletely spliced transcripts from the nucleus to the cytoplasm thus limiting their translation into aberrant polypeptides [23], [116], [117], [118], [119]. In addition to nuclear sequestration, transcripts that fail to complete splicing within biologically meaningful timeframes are eliminated [23], [113], [119].

This nuclear retention and elimination (NRE) pathway can function in combination with regulated intron splicing as a post-transcriptional gene regulation mechanism. Regulated IR events often occur in the ORF-encoding region, consistent with the overall enrichment of introns in this part of pre-mRNA. For example, retention of intron 3 in the ORF of mouse apolipoprotein E (ApoE) mRNA results is its efficient sequestration in the nucleus [120]. Regulation of human RBP SRSF1/ASF/SF2 expression levels relies in part on retention of an ORF-embedded intron that hinders nucleocytoplasmic export of the incompletely spliced transcripts [121]. Ptbp1-stimulated intron retention followed by NRE has been shown to prevent aberrant expression of several neuronal presynaptic proteins in non-neuronal cells [119]. Similar mechanisms control expression levels of the p53 inhibitor Mdm4 and several other proteins in response to defects in the core splicing machinery or DNA damage [122], [123].

Recent transcriptome-wide analyses suggest that IR is an exceptionally common form of AS in animals and plants committing large subsets of intron-containing mRNA isoforms to NRE or other forms of nuclear quality control [123], [124], [125]. However, it is worth noting that many intron-retained mRNAs appear to be efficiently exported to the cytoplasm where they may undergo NMD triggered by intronic PTCs (e.g., [126]; see Section 4.1) or localize to subcellular compartments specified by corresponding intron-encoded *cis*-elements (see Section 4.4).

### 4.4 ORF-specific mRNA localization elements

A majority of *cis*-elements controlling mRNA localization in the cell are thought to reside in the 3′UTR (see Section 3.4). However, several such elements have been identified in the ORF region and shown to be regulated by AS. For example, retention of intron 17a in the protein-coding region of the mRNA of calcium-activated big potassium channel (BKCa) targets this transcript to dendritic compartment and modulates excitability of hippocampal neurons through localized protein synthesis [127], [128].

More recently, a number of neuronal transcripts have been shown to contain unspliced introns enriched in SINE ID retroelements mediating dendritic localization [129], [130], [131]. However, it still remains to be investigated whether the ID-containing introns can be removed from localized mRNAs in the cytoplasm to enable production of full-length proteins [128], [129], [130], [132].

Another example is the surface receptor Robo3 regulating commissural axon midline crossing in developing nervous system [133]. The *Robo3* gene produces two alternative mRNA isoforms. Of these, the completely spliced Robo3.1 encodes a full-length Robo3 protein whereas the alternative Robo3.2 isoform retains intron 26 connecting exons 26 and 27 and gives rise to a truncated protein terminated by a PTC. Notably, Robo3.1 mRNA is constitutively translated before midline crossing inhibiting axon repulsion by the Robo ligand Slit. On the other hand, Robo3.2 mRNA is transported to the growth cone in a translationally silent form [134]. Upon midline crossing it undergoes a short burst of translation followed by rapid NMD degradation [126]. This generates small amount of Robo3.2 protein ensuring optimal repulsion of the contralateral part of the axon from the midline. An exciting aspect of this regulation circuitry is that intron 26 apparently contains *cis*-elements modulating mRNA localization, translational efficiency and stability.

ORF-encoded localization signals can also reside in alternative exons, as shown for the mRNA of the Stardust (Sdt) protein involved in the establishment of epithelial cell polarity during *Drosophila* embryogenesis [135]. This mRNA localizes to the apical side of the cell and the *cis*-element necessary and sufficient for this behavior is embedded within the coding cassette exon 3. Interestingly, inclusion of exon 3 diminishes during development, which results in robust apical targeting of Sdt mRNA at early stages of epithelial differentiation and its virtually uniform distribution in mature epithelial cells.

## 5 Conclusions and perspectives

Recent gene expression studies relying on deep sequencing in combination with more traditional molecular approaches have provided important insights into AS functions. We now know that most genes can generate more than one alternatively spliced transcript, substantially augmenting proteome complexity. AS also widely regulates mRNA stability, translation and localization. Blurring the classical division of mRNA into protein-coding and regulatory non-coding sequences, recent studies suggest that AS can expose or mask *cis*-regulation elements in the ORF region as well as in the 5′ and 3′UTRs.

The widespread occurrence of such “non-coding” functions of AS across eukaryotic domain and their occasional association with ultraconserved sequences (e.g., many alternative exons modulating NMD; see Section 4.1) argue that there is a strong evolutionary pressure to maintain this form of regulation. Do AS-dependent mechanisms offer any advantages compared to other forms of gene regulation, such as transcriptional control? We see several non-mutually exclusive possibilities that will be interesting to address in future studies. (1) Regulating mRNA behavior through AS may refine the “rough draft” of gene expression generated by the transcription machinery. This could be useful for example in differentiating gene expression outputs between distinct cell types originating from a common progenitor or between physiological states of the same cell. (2) AS may stabilize expression of target genes by linking their abundance with that of corresponding trans-acting factors. Indeed, many RBP regulators of AS are known to be expressed at relatively stable levels – at least in part due to post-transcriptional loops maintaining their own homeostasis and homeostasis of related RBPs (e.g., see Section 4.1). (3) Finally, AS (and post-transcriptional regulation in general) might change gene product concentrations more rapidly than it would be possible though purely transcriptional switches, which often involve time-consuming chromatin modification and initiation complex assembly steps. This would be especially valuable during rapid developmental transitions and in response to environmental cues.

We predict that ongoing analyses of high-throughput transcriptomics data will identify additional examples of non-coding roles for alternative pre-mRNA splicing events. Important challenges in this field include detailed mechanistic understanding of tissue- and cell-type specific AS and downstream regulation pathways including mRNA quality control and subcellular localization. There could be a considerable degree of crosstalk between distinct branches of AS-coupled post-transcriptional regulation (e.g.[126]) and further investigation of these molecular ties will likely result in exciting new discoveries. Akin to many other areas of life sciences, emerging technologies for rapid genome engineering and single-cell analyses will undoubtedly accelerate progress in this field bringing us closer to quantitative understanding of gene regulation mechanisms in developing systems.
